# Genome Sequence of *Bacillus coagulans* P38, an Efficient Polymer-Grade l-Lactate Producer from Cellulosic Substrates

**DOI:** 10.1128/genomeA.00495-15

**Published:** 2015-05-21

**Authors:** Lili Peng, Lifu Song, Lifan Sun, Yumeng Cai, Limin Wang, Bo Yu

**Affiliations:** a CAS Key Laboratory of Microbial Physiological and Metabolic Engineering, Institute of Microbiology, Chinese Academy of Sciences, Beijing, China; b Institute of Bioprocess and Biosystems Engineering, Hamburg University of Technology, Hamburg, Germany

## Abstract

*Bacillus coagulans* P38 is an efficient polymer-grade l-lactic acid producer from a cellulosic carbon source. Here, the draft 3.37-Mb genome sequence of this potential strain may provide useful information to further improve the strain performance for higher titers and, importantly, to understand the mechanism of its high tolerance for 2-furfural.

## GENOME ANNOUNCEMENT

Lactic acid is a valuable chemical that finds extensive use in the polymerization of lactic acid to polylactic acid. Many microorganisms, such as fungi, *Lactobacillus* species, and *Bacillus coagulans*, can produce lactic acid (1–3). Compared to the most frequently used lactic acid producers, such as *Lactococcus lactis* and *Lactococcus rhamnosus*, *B. coagulans* can grow optimally at 50 to 55°C, which is expected to minimize contamination in industrial-scale fermentations (4). Therefore, in recent years, there has been an interest in studies on the optical purity of l-lactic acid produced by this species (5, 6). *B. coagulans* strain P38 is an efficient producer of l-lactic acid, with a predominant capacity to tolerate up to 10 g/liter 2-furfural for lactic acid production. Its highly efficient production capability, combined with high inhibitor tolerance, indicates that *B. coagulans* P38 is a promising polymer-grade l-lactic acid producer from cellulosic biomass (6, 7).

Here, we present the draft genome sequence of *B. coagulans* P38 obtained by using the Illumina HiSeq 2000 system, which was performed by the Chinese National Human Genome Center at Shanghai, China. We obtained 9,253,431 high-quality-read base pairs with the Velvet program (8). Through the data assembly, we obtained 137 contigs, and the contig *N*_50_ is 57,692 bp. The average length of each contig is 25,385 bp, and the largest one is 272,938 bp, with a total length of 3.37 Mb.

The gene prediction was performed in line with the predicted results of Glimmer 3.02, GeneMark, and the Z-curve program software, with 3,720 genes were predicted. The G+C content of the predicted genes is 47.4%, and the average length of a coding sequence (CDS) is 765 bp. The maximum CDS length is 4,563 bp, and the minimum is 114 bp. Sixty tRNA sequences were found by tRNAscan, and 4 rRNA sequences were predicted via RNAmmer (9). Gene function annotations were made through searching the nucleotide collection, the KEGG protein database, and the SEED protein database of NCBI (10). The Clusters of Orthologous Groups (COG) classifications were performed using the Conserved Domains Database (CDD). The annotation result showed that 2,477 proteins have clear biological functions, of which 1,728 proteins have an ortholog of KEEG, and 2,434 proteins have a COG classification. All the matched homologous proteins are derived from 146 species, including *B. coagulans* 36D1 (11), with the highest percentage of 72.1%, followed by *B. coagulans* 2-6 (12), with a percentage of 20.1%.

*B. coagulans* P38 was predicted to possess complete metabolic pathways from the genome sequence analysis, including those for glycolysis, the tricarboxylic acid cycle, and the pentose phosphate pathway. One l-lactate dehydrogenase gene and one d-lactate dehydrogenase gene were identified from the genome. A possible lactate/malate dehydrogenase gene was also annotated. Several short-chain dehydrogenase and alcohol dehydrogenase genes were annotated from the genome sequence, which might provide useful information to investigate the 2-furfural tolerance mechanism (13).

### Nucleotide sequence accession numbers. 

This whole-genome shotgun project has been deposited at DDBJ/EMBL/GenBank under the accession no. JSVI00000000. The first version (JSVI01000000) is described in this paper.
